# Does Hysteroscopic Dissection of Partial Uterine Septum Represent a Risk Factor for Placental Abnormalities in Subsequent Pregnancy Compared with Controls Undergoing Other Hysteroscopic Surgery? Results from a Large Case–Control Analysis

**DOI:** 10.3390/jcm12010177

**Published:** 2022-12-26

**Authors:** Pia Hajšek, Gaetano Riemma, Sara Korošec, Antonio Simone Laganà, Vito Chiantera, Mislav Mikuš, Helena Ban Frangež

**Affiliations:** 1 Faculty of Medicine, University of Ljubljana, 1000 Ljubljana, Slovenia; 2 Obstetrics and Gynecology Unit, Department of Woman, Child and General and Specialized Surgery, University of Campania “Luigi Vanvitelli”, 80128 Naples, Italy; 3 Department of Human Reproduction, Division of Gynaecology and Obstetrics, University Medical Centre Ljubljana, 1000 Ljubljana, Slovenia; 4 Unit of Gynecologic Oncology, ARNAS “Civico-Di Cristina-Benfratelli”, Department of Health Promotion, Mother and Child Care, Internal Medicine and Medical Specialties (PROMISE), University of Palermo, 90127 Palermo, Italy; 5 Department of Obstetrics and Gynecology, Clinical Hospital Center Zagreb, 10000 Zagreb, Croatia

**Keywords:** congenital uterine anomalies, uterine septum dissection, placental abnormalities, neonatal outcome, IVF/ICSI, hysteroscopy, complications

## Abstract

Background: Hysteroscopic septum dissection (HSD) is thought to improve fertility and pregnancy outcomes. However, the available literature suggests that uterine surgery can cause placental abnormalities in subsequent pregnancies. Methods: A case–control study was performed at the University Medical Center of Ljubljana, Department of Human Reproduction, from 1 January 2016 to 31 December 2018. The primary outcome was the association between HSD and the occurrence of placental abnormalities. We included women who underwent HSD due to infertility. Age-matched women who underwent hysteroscopic surgery for other issues were considered as controls. In addition, we divided the groups according to conception method. Only singleton pregnancies and first delivery were considered. Results: A total of 1286 women (746 who underwent HSD and 540 controls) were included in the analysis. HSD had no influence on placental abnormalities since the ratio was comparable regardless of the method of conception (113/746 vs. 69/540; *p* = 0.515). Infertile women who conceived naturally after HSD had a normal placentation rate comparable to women who did not undergo HSD (380/427 vs. 280/312; *p* = 0.2104). The rate of placental abnormalities in women who achieved pregnancy with IVF/ICSI procedures following HSD was comparable to that of women who did not undergo HSD (52/319 vs. 33/228; *p* = 0.5478). Placenta previa occurred significantly more often in infertile women without HSD after IVF/ICSI compared to natural conception (2/312 vs. 7/228; *p* = 0.0401). Conclusions: HSD was not associated with higher rate of placental abnormalities in the first singleton pregnancy compared with other hysteroscopic procedures. A higher rate of placenta previa in pregnancies following IVF/ICSI procedures, which was shown by our research, is corroborated by previous research findings.

## 1. Introduction

Septate uterus is the most common congenital uterine anomaly [1,2]. It results from incomplete resorption of medial part during development of the female reproductive system [3].

The impact on fertility and pregnancy outcomes of a partially or complete septate uterus are still debated. Several studies and reviews suggest that the presence of uterine septum presents an increased risk for subfertility [4,5,6,7,8], spontaneous abortions [4,5,6,7,8,9], preterm delivery [5,6,7,8], fetal growth restriction [8], cesarean delivery [6], placental abruption [6,8], and fetal malpresentation [5,6,8], as well as a decreased live birth rate [6,7,9] and higher perinatal mortality [8], regardless of the method of conception. Even though the exact extent to which septate uterus affects the fertility of women is still unknown, it remains a clear cause of concern, as the diagnosis usually follows a previous diagnosis of subfertility or numerous pregnancy losses [10].

Hysteroscopic septum dissection (HSD) as a method to improve reproductive performances of infertile women is still not universally accepted, as evidence on its effectiveness is mainly based on retrospective studies [1,8,10,11]. According to several studies, the procedure may increase the probability of natural conception in infertile women [12,13,14,15,16,17]. Many studies have found that correction of septate uterus can improve pregnancy outcomes [7,8,13,15,16,17,18]. HSD is often performed as a part of infertility workup, before the assisted reproduction technique (ART) protocol, because it is believed to raise the success rate of in vitro fertilization (IVF)/intracytoplasmic sperm injection (ICSI) procedures [4,9,19]. In contrast, there are studies suggesting that HSD is not beneficial to improve reproductive outcome [2,20]. HSD is generally considered to be a safe option of treatment when performed by a skilled surgeon, and it is an established method of treatment in women with recurrent miscarriages and preterm births, although several authors agree that it is also beneficial for infertile women [4,9,12,14,15,16,17,19].

As with any procedure, complications may occur. There is a scarce amount of data on obstetric complications of women who were subjected to HSD in subsequent pregnancies, in contrast to the large number of studies on the impact of HSD on reproductive outcome. Because of previous findings suggesting that hysteroscopic surgery is generally a risk factor for the development of placental abnormalities [21,22,23,24], the aim of this study was to determine the rate of placental abnormalities after HSD compared with that after other types of hysteroscopic procedure.

## 2. Materials and Methods

We designed a retrospective case–control study on infertile women who gave birth to singletons after infertility treatment at the University Medical Centre of Ljubljana, Department of Human Reproduction, from 1 January 2016 to 31 December 2018.

We included in the study group (cases) reproductive-aged women (aged between 18 and 41 years old) who had a three-dimensional (3D) ultrasonographic diagnosis of partial uterine septum (class U2a according to the European Society of Human Embryology (ESHRE) and European Society of Gynecological Endoscopy (ESGE) classification of female genital tract congenital anomalies) and had a history of primary infertility or secondary infertility and early pregnancy loss. According to the World Health Organization definition, women were considered infertile whenever they failed to conceive naturally after 1 year of regular unprotected intercourse. Partners’ semen was analyzed, and women underwent ultrasound for diagnosing other possible uterine pathologies.

Women were excluded from the study group in case of any other endocrine, autoimmune, metabolic, oncological, and/or severe systemic diseases, as well as in the case of dysmorphic uterus (class U1a or U1b), complete septate uterus (class U2b), Müllerian anomaly other than partial uterine septum (class U2a), and/or other gynecological disease. The decision to include only partial uterine septum (class U2a) was made to avoid any potential bias and reduce heterogeneity of the study group, considering also that complete uterine septum (class U2b) is significantly less frequent than partial uterine septum (class U2a) [5].

Infertile women in the study group underwent HSD, while infertile women who underwent hysteroscopic procedures (endometrial biopsy, endometrial polypectomy, or myomectomy) other than HSD were considered controls. In order to ensure consistency of the data analysis and avoid potential biases, we also excluded from the control group women affected by any other endocrine, autoimmune, metabolic, oncological, and/or severe systemic diseases, as well as any gynecological disorder other than the indication to undergo the hysteroscopic procedure.

Additionally, enrolled women were divided into subgroups according to method of conception in subsequent pregnancy: IVF/ICSI vs. natural conception.

In cases of normal ultrasound and confirmed male partner infertility, women were referred to IVF/ICSI procedures. If partial septate uterus was found on transvaginal 3D ultrasound, HSD was performed aiming to enhance pregnancy rates in IVF/ICSI procedures. In cases where the partner’s semen was normal, women had diagnostic or operative laparoscopy and/or hysteroscopy as scheduled for suspect of organic pathology or tubal factor infertility. If pregnancy was not achieved, they were referred to IVF/ICSI procedures.

HSD was performed at University Medical Center of Ljubljana, Department of Human Reproduction. The patients received misoprostol vaginally to soften and dilate the cervix, 2 h before hysteroscopy, which was performed under general anesthesia. Uterine septum dissection was performed with a 22 Fr monopolar resectoscope (Karl Storz, Tuttlingen, Germany). An electrosurgical incision the uterine septum was made between the anterior and posterior uterine walls, up high into the uterine fundus, until the space between the tubal ostia was in a straight line. Hysteroscopic surgery rather than septum dissection (endometrial biopsy, endometrial polypectomy, or myomectomy) was also performed with a 22 Fr monopolar resectoscope (Karl Storz, Tuttlingen, Germany), in accordance with common clinical practice of our institution.

The design, analysis, interpretation of data, drafting, and revisions conform to the Helsinki Declaration, the Committee on Publication Ethics (COPE) guidelines, and the Reporting of Studies Conducted using Observational Routinely Collected health Data (RECORD) statement, available through the Enhancing the Quality and Transparency of Health Research (EQUATOR) network. The data collected were anonymized, considering the observational nature of the study, without personal data that could lead to formal identification of the patient. Each patient enrolled in this study was informed about the procedures and signed consent to allow data collection and analysis for research purposes. The study was approved by the Medical Ethics Committee of the Republic of Slovenia (approval ID: 0120-174/2018/6). The data from deliveries were gathered from National Perinatal Information system of Slovenia (NPIS). No remuneration was offered to the patients to give consent to be enrolled in this study.

We aimed to observe the influence of two main independent variables: HSD and method of conception (natural conception or IVF/ICSI procedures). Independent covariables assessed were the following: age of woman at delivery, previous early pregnancy loss, previous surgical termination of pregnancy and previous ectopic pregnancy.

The main observed dependent variables in the study were placental abnormalities, such as placental abruption, placenta previa, adherent placenta (defined as placentas that had to be manually removed after the delivery, but with no abnormal placentation found), incomplete placenta, placental calcification, placental infarction, placenta accreta spectrum (PAS), and vasa previa. Moreover, pregnancy-related dependent variables were also assessed: mean gestational age at delivery, birth weight, and APGAR score after 1 and 5 min.

For IVF/ICSI conceived pregnancies, the gestational age was determined from the day of oocyte retrieval or embryo transfer. According to clinical standards, the gestational age was determined by measuring the fetal crown–rump length in the first trimester for naturally conceived pregnancies.

### Statistical Analysis

Statistical analysis was performed using IBM SPSS Statistics, version 25 (IBM Corp., Armonk, NY, USA). Different statistical methods were used to test for differences between observed variables, depending on the type of variable. Fisher’s exact test or chi-square test was used for the analysis of categorical variables, whereas the analysis of variance (ANOVA) test or Kruskal–Wallis test was used for continuous variables. Differences between variables were considered statistically significant if the *p*-value (*p*) was <0.05.

## 3. Results

**Figure 1:**
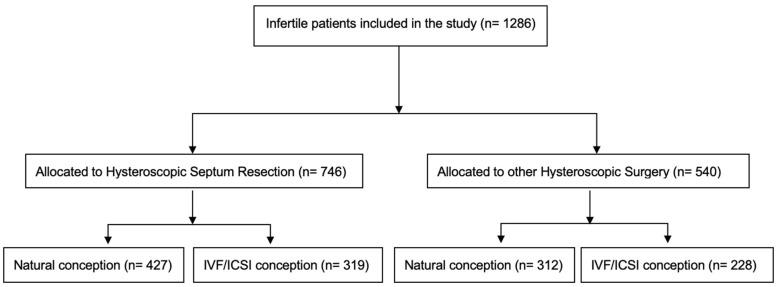
Flowchart of patients included in the analysis.

A total of 1286 women were included in the analysis. The study group included 746 infertile women who underwent HSD; among them, 427 conceived naturally and 319 conceived with IVF/ICSI procedures. The control group consisted of 540 women who underwent other hysteroscopic procedures; 312 conceived naturally and 228 conceived with IVF/ICSI procedures (Figure 1).

Women who conceived with IVF/ICSI were significantly older (31.72 ± 4.39 and 31.71 ± 3.70) than those who conceived naturally (30.84 ± 4.21 and 30.68 ± 4.12), unrelated to the presence of a septate uterus (*p* = 0.024 and *p* = 0.028, respectively). Women who underwent HSD had statistically more previous early pregnancy losses than those with normal uterus, showing no relationship with the subsequent method of conceiving (0.438 ± 0.789 vs. 0.311 ± 0.706; *p* = 0.016 and 0.376 ± 0.720 vs. 0.211 ± 0.522; *p* = 0.008) (Table 1).

For all the included women, time between surgery and pregnancy was 15.0 ± 3.0 months, with no differences between the HSD and control groups. Women who underwent other hysteroscopic surgery and who conceived with IVF/ICSI had more previous early pregnancy losses (0.048 ± 0.214 vs. 0.145 ± 0.461; *p* = 0.024) and ectopic pregnancies (0.003 ± 0.057 vs. 0.039 ± 0.195; *p* = 0.008) than those who conceived naturally (Table 1). Regardless of the method of conception, women after HSD had a comparable ratio of normal placentation as controls who underwent other hysteroscopic surgery. Similarly, the overall rate of placental abnormalities was comparable between HSD and other hysteroscopic procedures [14.61% (109/746) vs. 11.85% (64/540); *p* = 0.16] (Table 2).

There were no significant differences in the occurrence of placental abnormalities among the 427 women after HSD that conceived naturally and the 319 women who later conceived with IVF/ICSI (57/427 vs. 52/319; *p* = 0.2949). The comparison of the various placental abnormalities in 312 controls that underwent other hysteroscopic surgery and conceived naturally or with IVF/ICSI also showed no differences (Table 2), except for placenta previa in the control group, which was more common in post-IVF/ICSI pregnancies of women in the control group compared to women that conceived naturally (7/228 vs. 2/312; *p* = 0.0401).

We observed a significant difference in mean gestational age between the control group with natural conception, which was few days longer, compared to pregnancies in the same group after IVF/ICSI (Table 2: 38.98 ± 1.847 vs. 38.38 ± 2.760; *p* = 0.029). This difference was not observed in women treated with HSD (Table 3).

## 4. Discussion

HSD is a common operative procedure often performed in women with early pregnancy loss and preterm birth, as well as in the infertile population, since it improves fertility rates in natural conception [1,2,3,4,5,6], as well as in Assisted Reproduction Technology (ART) procedures [7,8,9].

While several studies have investigated the effects of uterine septum and its treatment on reproductive outcomes, very few studies have investigated pregnancy complications following hysteroscopic intervention in the uterus, specifically after HSD. The complications that arise during and immediately after HSD are better studied, and they include bleeding [10], fluid overload with a distension agent [11], intrauterine postoperative adhesions [12], and uterine perforation during the procedure [13].

There is certain evidence suggesting that HSD may be associated with complications during subsequent pregnancy. Uterine rupture has been shown to be a very rare complication that can occur even without prior uterine perforation during HSD [14]. Some authors suggest that women after HSD still have an increased risk of fetal malpresentation, low birth weight, and cesarean delivery [15]. Additionally, studies regarding the impact of uncomplicated hysteroscopic interventions on pregnancy outcomes have been conducted [16,17], due to the assumptions that tissue damage during cervical dilatation is a risk factor for cervical prolapse, leading to preterm birth [18]. To date, robust data suggest that hysteroscopic procedures in infertile women cannot be considered risk factors for preterm birth [16,17].

Baldwin et al. highlighted that hysteroscopic interventions are a risk factor for placenta accreta [19]. The possible association of hysteroscopic interventions with placental abnormalities was also indicated by other authors who found a higher incidence of placenta accreta and placenta previa in women after hysteroscopic treatment of intrauterine adhesions [20] and hysteroscopic myomectomy [21,22].

However, we found that HSD is not associated with a higher risk of developing any placental abnormality compared with other hysteroscopic procedures. The total rate and rates of individual placental abnormalities after HSD, regardless of natural conception or with IVF/ICSI procedures, were comparable to rates in those who underwent other hysteroscopic surgery. Our results are consistent with the findings of a comparative study by Kenda-Suster et al. [23], which investigated the incidence of placenta previa, placental abruption, and retained and adherent placenta in infertile women and women with recurrent pregnancy loss after HSD. Our findings are also in line with results of study of Moffat et al. [17] that concluded that hysteroscopic interventions in the uterus in infertile women were not a risk factor placenta previa. In our research, we additionally analyzed the incidence of placenta accreta, vasa previa, placental calcification, and placental infarction and found that they do not occur significantly more often after HSD than after other hysteroscopic procedures.

With the development of ART procedures, the importance of operative treatment of infertility is becoming questionable [24]. In addition, operative treatment is not always successful, and ART remains a first-line treatment for couples in whom operative treatment did not lead to the desired result, as well as in women with bilateral tubal factor infertility or in cases of a severe form of male infertility [7]. Accumulating evidence suggests that IVF/ICSI procedures are an important risk factor for pregnancy complications, including placental abnormalities [25,26,27]. Therefore, we divided the subjects into subgroups according to method of conception to assess its influence on placental abnormalities.

The results of our study showed that there is a significant difference in the incidence of placenta previa between subgroups of infertile women without HSD who conceived naturally relative to patients with IVF/ICSI. Our results are consistent with the findings of other authors, who reported that placenta previa is more common after IVF/ICSI procedures [25,27]. Among the subgroups of women who underwent HSD, the difference in the incidence of placenta previa according to the mode of conception was not found to be statistically different.

Some studies suggested that placental abruption [28], vasa previa [29], other PAS diseases [30,31], and retained placenta [32,33] were more common in IVF/ICSI pregnancies. Women with septate uterus had, as expected, statistically significantly more early pregnancy losses before undergoing HSD than infertile women with a normal uterus. Our results are in line with many studies that demonstrated the negative impact of uterine septum on pregnancy outcome, including higher rates of early pregnancy losses [7,9,33,34,35,36,37].

However, several limitations should be taken into account for a proper data interpretation of our study. Firstly, our research was retrospective, and this decreases the quality of the investigated evidence. Secondly, we included only partial uterine septum (class U2a), excluding complete uterine septum (class U2b) from the study group. On the one hand, this reduced heterogeneity of the data analysis; on the other hand, this limitation may prevent to generalize our findings for women affected by complete uterine septum (class U2b). From this perspective, future investigations including complete septum could be considered a research priority in this field. Thirdly, 3D ultrasound was not performed about 8–12 weeks after surgery in order to ensure there was no remaining relevant septum. Fourthly, we included women with both primary and secondary infertility, which could have affected the interpretation of the results. To date, there has been only one recent randomized clinical trial (RCT) comparing women who underwent HSD vs. expectant management [38], suggesting the inefficacy of HSD in improving reproductive outcomes. However, data available for our study were acquired before the publication of Rikken et al.’s trial [38]; for this reason, the clinical practice related to our study was based on the evidence available before the abovementioned study [36]. Nonetheless, it is indeed notable that a control group based of women with a septate uterus not undergoing HSD would have been more appropriate; however, since the literature available at the beginning of our study somewhat showed a benefit in terms of feasibility and pregnancy outcome with HSD, it was considered unethical to deprive women of a way to increase pregnancy chances. In addition, it would have been appropriate to compare the rate of placental abnormalities in women who underwent HSD and in women who did not undergo any hysteroscopic surgery, in order to obtain a more robust data analysis of the topic. For this reason, our study was specifically designed to evaluate the rate of placental abnormalities after HSD compared with other types of hysteroscopic procedures. Therefore, an additional limitation is related to the fact that the overall results of our study may be interpreted differently upon accumulating more updated evidence on the clinical efficacy of HSD. However, the meticulous research protocol, the consistency of evaluated outcomes, and the large sample size could be considered strengths of our study.

## 5. Conclusions

HSD was not associated with a higher rate of placental abnormalities in the first singleton pregnancy compared with other hysteroscopic procedures. As a corollary result, our study also confirmed the earlier findings that IVF/ICSI procedures are associated with an increased rate of placenta previa. We take the opportunity to solicit further research in order to compare the rate of placental abnormalities in women who underwent HSD and women with uterine septum who did not undergo HSD, investigating also whether the type of uterine septum (partial or complete) may affect this outcome.

## Figures and Tables

**Table 1:** Clinical characteristics of patients.

	HSD		without HSD		*p*-Value			
	Conception							
	Natural (A)	IVF/ICSI (B)	Natural (C)	IVF/ICSI (D)	AC	BD	AB	CD
Number of patients	427	319	312	228				
Mean age (mean years ± SD)	30.84 ± 4.21	31.72 ± 4.39	30.68 ± 4.12	31.71 ± 3.70	1	1	0.024 *	0.028 *
Primary vs. secondary infertility (%)	285 vs. 142 (66.7% vs. 33.3%)	225 vs. 94 (70.5% vs. 29.5%)	210 vs. 102 (67.3% vs. 32.7%)	165 vs. 63 (72.4% vs. 27.6%)	0.872	0.640	0.271	0.207
Previous early pregnancy losses cases (mean ± SD)	0.438 ± 0.789	0.376 ± 0.720	0.311 ± 0.706	0.211 ± 0.522	0.016 *	0.008 *	1	0.788
Previous ectopic pregnancies cases (mean ± SD)	0.021 ± 0.144	0.056 ± 0.302	0.003 ± 0.057	0.039 ± 0.195	0.152	1	0.448	0.008 *

* *p* < 0.05; HSD: hysteroscopic septum dissection; IVF: in vitro fertilization; ICSI: intracytoplasmic sperm injection.

**Table 2:** Comparison of placental abnormalities in infertile women.

	HSD		without HSD		*p*-Value			
	Conception							
	Natural (A)	IVF/ICSI (B)	Natural (C)	IVF/ICSI (D)	AC	BD	AB	CD
Placental abruption	8 (1.9%)	2 (0.6%)	4 (1.3%)	2 (0.9%)	0.7697	1	0.2020	1
Placenta previa	5 (1.2%)	4 (1.3%)	2 (0.6%)	7 (3.1%)	0.7050	0.2149	1	0.0401 *
Adherent placenta	9 (2.1%)	14 (4.4%)	6 (1.9%)	7 (3.1%)	1	0.5034	0.0878	0.4083
Incomplete placenta	19 (4.4%)	17 (5.3%)	12 (3.8%)	11 (4.8%)	0.7151	0.8463	0.6074	0.6675
Placental calcification	14 (3.3%)	9 (2.8%)	10 (3.2%)	4 (1.8%)	1	0.5722	0.8318	0.4132
Placental infarction	4 (0.9%)	5 (1.6%)	1 (0.3%)	1 (0.4%)	0.4034	0.4086	0.5078	1
PAS	2 (0.5%)	0 (0.0%)	1 (0.3%)	0 (0.0%)	1	1	0.5098	1
Vasa previa	0 (0.0%)	1 (0.3%)	0 (0.0%)	1 (0.4%)	1	1	0.4276	0.4222
Preeclampsia	11 (2.5%)	12 (3.7%)	8 (2.6%)	8 (3.5%)	0.593	0.534	0.237	0.347
No placental defect	370 (86.7%)	267 (83.7%)	280 (89.7%)	196 (86.0%)	0.2104	0.5478	0.2949	0.1812

* *p* < 0.05; HSD: hysteroscopic septum dissection; IVF: in vitro fertilization; ICSI: intracytoplasmic sperm injection; PAS: placenta accreta spectrum.

**Table 3:** Comparison of neonatal outcome in subsequent pregnancies after infertility treatment.

	HSD		without HSD		*p*-Value			
	Conception							
	Natural (A)	IVF/ICSI (B)	Natural (C)	IVF/ICSI (D)	AC	BD	AB	CD
Mean gestation age (weeks ± SD)	38.78 ± 2.451	38.34 ± 2.776	38.98 ± 1.847	38.38 ± 2.760	1	1	0.091	0.029 *
Birth weight (g ± SD)	3256 ± 601	3219 ± 678	3352 ± 530	3241 ± 684	0.237	1	1	0.243
APGAR after 1 min	8.76 ± 1.011	8.70 ± 1.292	8.80 ± 1.026	8.75 ± 1.131	1	1	1	1
APGAR after 5 min	9.07 ± 0.854	9.04 ± 0.951	9.12 ± 0.859	9.09 ± 0.925	1	1	1	1

* *p* < 0.05; HSD: hysteroscopic septum dissection; IVF: in vitro fertilization; ICSI: intracytoplasmic sperm injection.

## Data Availability

The data presented in this study are available on reasonable request from the study coordinator (H.B.F.).
